# Reshaping healthcare with wearable biosensors

**DOI:** 10.1038/s41598-022-26951-z

**Published:** 2023-03-27

**Authors:** Aaron Asael Smith, Rui Li, Zion Tsz Ho Tse

**Affiliations:** 1 College of Engineering, University of Georgia, Athens, GA 30602 USA; 2 Tandon School of Engineering, New York University, New York, NY 11201 USA; 3 Department of Engineering and Material Science, Queen Mary University of London, London, E1 4NS UK

**Keywords:** Electrical and electronic engineering, Quality of life

## Abstract

Wearable health sensors could monitor the wearer's health and surrounding environment in real-time. With the development of sensor and operating system hardware technology, the functions of wearable devices have been gradually enriched with more diversified forms and more accurate physiological indicators. These sensors are moving towards high precision, continuity, and comfort, making great contributions to improving personalized health care. At the same time, in the context of the rapid development of the Internet of Things, the ubiquitous regulatory capabilities have been released. Some sensor chips are equipped with data readout and signal conditioning circuits, and a wireless communication module for transmitting data to computer equipment. At the same time, for data analysis of wearable health sensors, most companies use artificial neural networks (ANN). In addition, artificial neural networks could help users effectively get relevant health feedback. Through the physiological response of the human body, various sensors worn could effectively transmit data to the control unit, which analyzes the data and provides feedback of the health value to the user through the computer. This is the working principle of wearable sensors for health. This article focuses on wearable biosensors used for healthcare monitoring in different situations, as well as the development, technology, business, ethics, and future of wearable sensors for health monitoring.

## Introduction

As the worldwide aged population has grown in recent years, the medical sector has been confronted with a new challenge: how to assist the patients in achieving health monitoring. Biosensors that enable real-time health monitoring, prevention, and treatment have been the emphasis in recent years. As smart wearable devices have become more popular, they can now be used to predict disease, and people improving their health beyond exercise through wearable devices will become a new trend^1^. Wearables are a burgeoning market, and their capabilities have evolved from tracking steps to monitoring medical issues and enhancing general health^2^.

**Figure 1:**
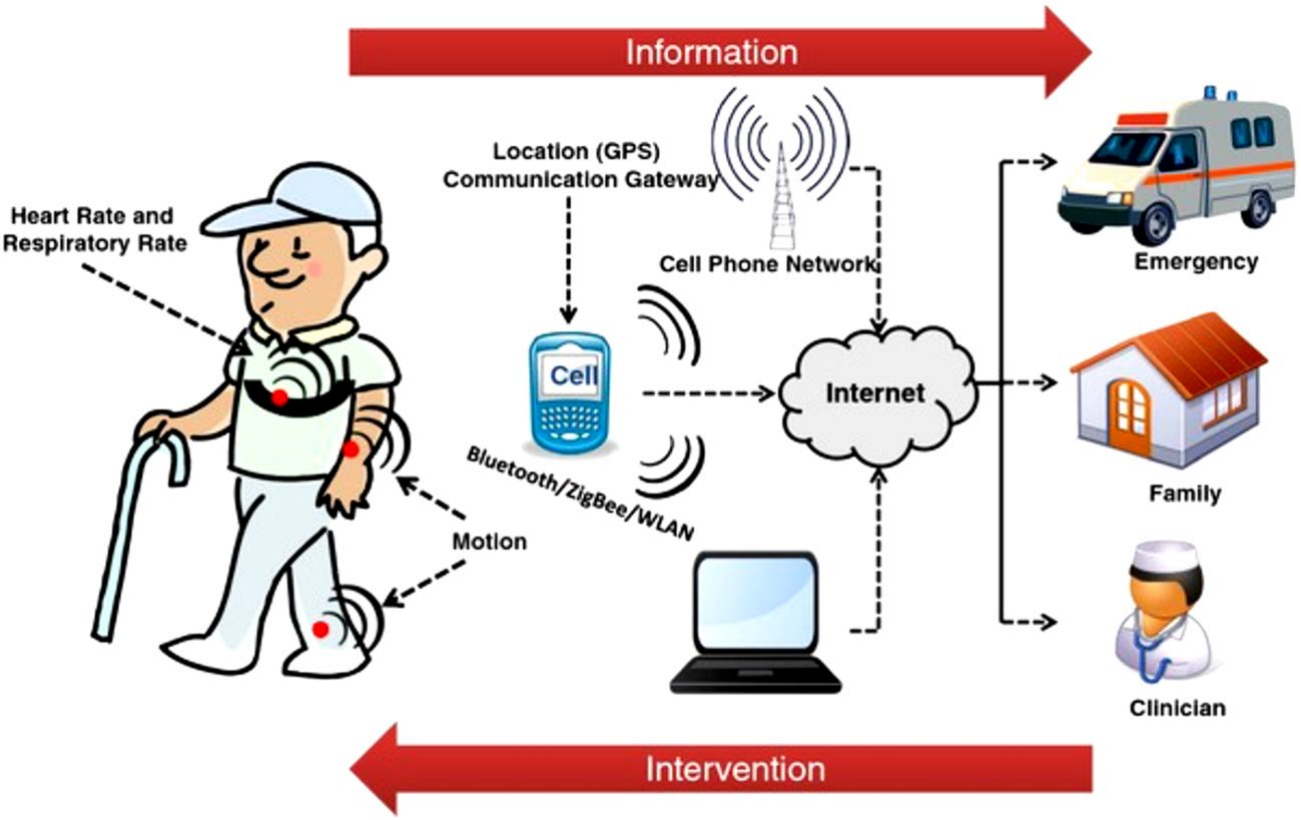
Schematic diagram of a remote health monitoring system based on wearable sensors^3^. Data on physical health is collected through the sensors worn and transmitted to healthcare workers through networked devices. Healthcare workers can use this data to take an action.

Figure 1 shows a conceptual demonstration of a remote monitoring system, from which wearable sensors are used to collect data on the wearer's physiological indicators to monitor the patient's own physical condition in real-time. The patient's data is passed on to the mobile device through wireless communication, and then the data is passed to the remote center via the Internet. When the system detects a patient's sudden condition, such as a fall, it sends an alert message to the emergency service center to provide timely assistance to the patient. In an emergency, the data-linked family members and caregivers are also alerted to each other to let the relevant personnel know the status of the current patient.

The major purpose of these sensors is to assist users in monitoring their own health and to provide timely warnings to customers who are ill. Wearable sensors have the main advantage of allowing consumers to conduct scientific health monitoring anywhere and at any time. Because the user visits the clinic for a diagnostic and the doctor utilizes the external sensor to make a diagnosis in a specific location, the user can save a lot of time if the portable sensor is worn. The advantages of wearable sensors for users are more prevalent, especially for users who use a range of portable sensors. For patients with chronic diseases, wearable devices can reduce the number of hospitalizations, because they can adjust their daily routines and diets in time according to the display of device data, which can save expensive treatment costs. Wearables can replace medical gear such as ECG gadgets and defibrillators by precisely measuring body temperature and heart health, which can help hospitals save money on equipment^3^. In addition, wearable devices can help patients better manage their lives and health, so that patients can understand their current physical condition in real-time through the device so that they can be hospitalized in a timely manner. People are paying more attention to their own health as their living standards improve, thus people’s attention to the heart, blood oxygen monitoring, sports and fitness, and related equipment has expanded dramatically^4^.

Sensor detection for a variety of chronic and acute disorders has been studied since the nineteenth century. The pacemaker is the first sensor for monitoring human health by sending electrical impulses to the heart to allow it to beat more consistently^5^. Humans began to utilize wearable sensors for health purposes as early as 1960^6^. According to research by Jeffrey and Parsonnet, the first pacemaker was implanted in a 40-year-old patient in 1958 by thoracic surgeon Erik Senning of Karolinska Hospital in Stockholm, who also implanted a pulse generator with rechargeable NiCd batteries^6^. The pacemaker's progress much like other devices has been amazing and swift.

People are becoming more enthusiastic about the future of health-related sensors. Different wearable health sensors^7–9^ and implantable sensors^10–12^ as well have been created and utilized since then. When the sensor is functional, it is important to know how to acquire the patient's health information and new technology with phone apps are helping make that possible.

In recent years, the face of wearables has shifted dramatically, with researchers shifting their attention from tracking people's regular exercise routines to addressing important difficulties in healthcare applications such as diabetic patient management and geriatric remote monitoring. To meet these big obstacles, scientists have focused their efforts on developing wearable biosensors, which are defined as sensing devices that incorporate biometric aspects into sensor operations, such as enzymes, antibodies, cell receptors, or organelles.

The sensor unit, first and foremost, is a required feature of all modern smart gadgets and comprises of many circuit boards that assemble sensors. The data collected by the sensors is sent to a central processing module, such as a personal digital assistant (PDA). The acceleration sensor is mounted on a pair of pants, and the circuit board is connected to a laptop in Laerhoven and Cakmakci's experiment. The circuit board will send data from the acceleration sensor on the user's pants to the laptop when the user is exercising^13^. For a long time, Lee and Mase have used sensor units to detect user behavior; they use a gyroscope, an angular velocity sensor, and an acceleration sensor to detect user behavior; these sensors are connected to a Linux PDA^14^, which is a sensor unit; the hardware for data processing is smaller than that of a notebook, and meets the standard of portability of the monitoring system and does not interfere with normal life which places a crucial role in sensor device design.

Sensors can be physically attached to the body, via avenues of standard clothing, watches, mobile phones, and other items that can be embedded with sensors in daily life. Flexible sensors are these types of sensors, and today's advances in printed electronics and materials allow some tiny sensors to be worn as skin patches^15^. These biosensors can measure skin conductance, heart rate, and body temperature, as well as detect changes in pH, glucose, and salt in the human body^16,17^ (Table 1).

**Table 1:** Sensing capabilities commonly found in wearable devices.

Sensor	Description	Privacy invasiveness
^26^Accelerometer x	Measures the acceleration force that is applied to a device	Low
^26^Magnetometer x	Measures the geomagnetic field strength	Low
^26^Gyroscope x	Measures a device’s rate of rotation around each of the three physical axes (x, y, and z)	Low
^27^Ambient light	Measures ambient light level	Low
^28^Proximity x	How far away an object is from the phone’s screen	Low
^26^Touch state x	Records movement, pressure, and size of screen touch interaction	Medium
^29^Screen state	Records every time the screen is turned on—off	Medium
^30^Video	Captures video and pictures	High
^26^GPS x	Provides user location coordinates	High
^28^Wi-Fi x	Provides data about the BSSID and signal strength of the nearby Wi-Fi access points	High
^31^Cell towers	Provides information aboutthe nearby cellphone towers	High
^28^Bluetooth x	Detects nearby Bluetooth capable devices	High
^28^Ambient temperature x	Measures the ambient room temperature	Low
^26^Pressure x	Measures the ambient air pressure	Low
^32^Galvanic Skin Response	Measures electrical conductance of the skin	Medium
^26^Electrocardiogram x	Measures heartrate activity	Medium
^28^Skin temperature x	Measures the temperature of the skin	Medium

Smart gadgets can be used to track data with greater accuracy, thanks to advancements in miniaturization technology. They can be woven into clothing as in-textile electronics, implanted into our everyday necessities, or even buried in the human ear. One of the most promising areas for wearable healthcare technologies right now is remote patient monitoring^18^. Wearable smart goods have also evolved from counting steps to monitoring physical health, providing healthcare staff with vital information such as blood pressure readings and potential arrhythmias.

Regarding smartphones, their role has expanded to include not only monitoring health but also carrying out simple, right, and healthy life interventions for users^19,20^. Some programs can also provide simple remedies^21^, such as mental diseases, because of the high frequency of daily use of smartphones and the high degree of user dependence. According to market research firm Counterpoint, the smartwatch market's future development focus is still fitness and health applications, therefore blood oxygen monitoring functions are common in existing wearable devices^3,22^. On another note, breast cancer has always been the leading cause of cancer death in women, and Cyrcadia Health, an American medical biosensor company, has been working with Jabil Medical Technology to develop a bra that can detect breast cancer, hoping to provide users with an early diagnosis of breast cancer. The application of this healthcare technology also marks another trend of change in wearable devices, from wrist-worn devices to body-wearable devices. Smart clothing built for specific conditions has been shown to be more practical, comfortable, durable, and reliable than smartwatches or bracelets^23^. Smart clothing built for specific conditions has been shown to be more practical than smartwatches and bracelets as certain demographics of the population, including children, the elderly, and individuals with chronic illnesses as well as mental health disorders, may find electronically embedded clothing to be especially helpful. Clothing could be designed ergonomically to fit the wearer’s body shape; the functionality could be maximized without negatively intrude the user’s personal space^24^.

**Figure 2:**
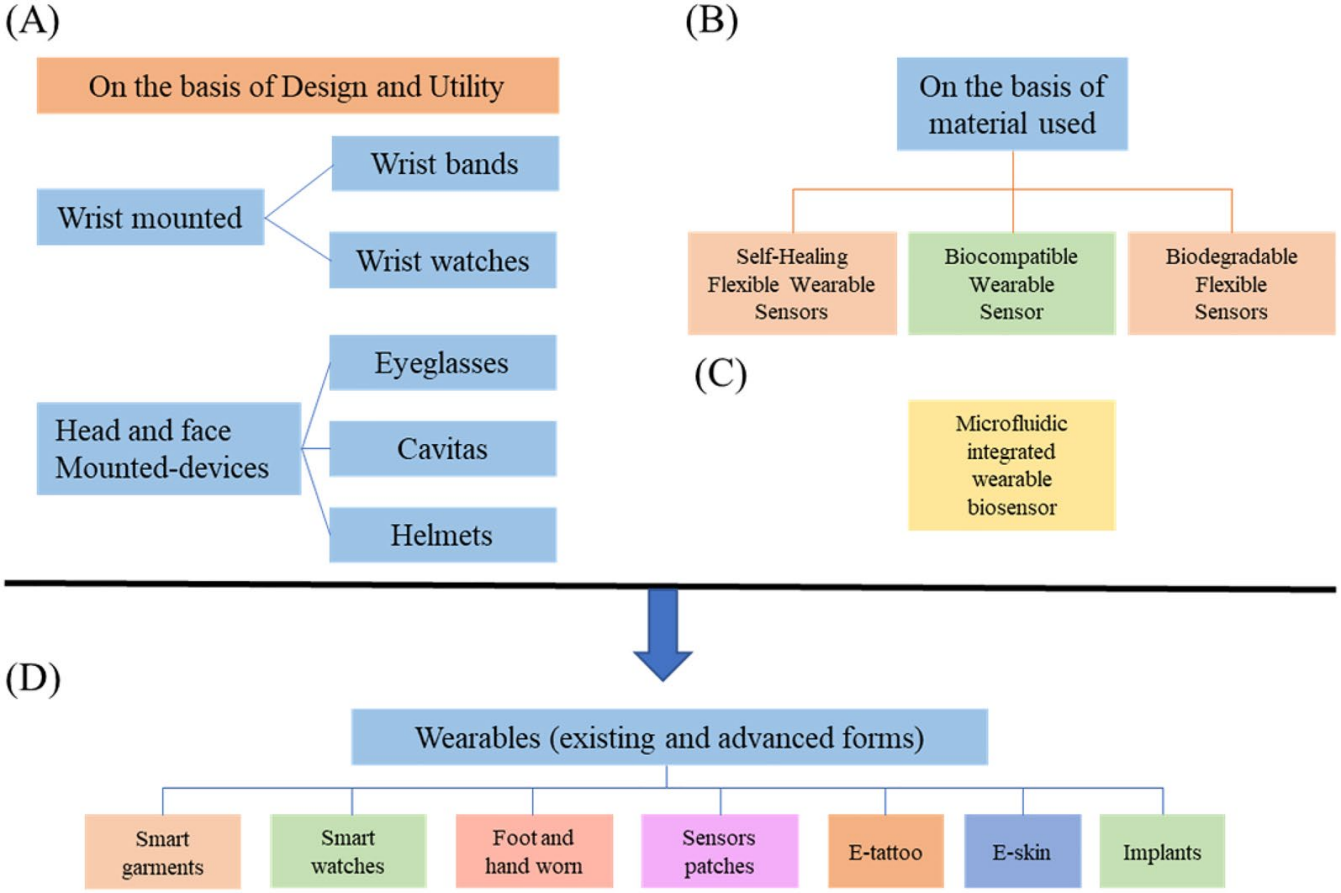
Application principles and categorical examples of wearable biosensors^25^.

Figure 2 shows the thought process of developing wearable devices and their sensor designs. The figure illustrates seven potential types of wearable devices ranging from smart garments to implants and these devices are all dependent on the sensor device design. Every psychological signal has a different sensor that can detect and measure the signal; therefore, the sensor design application will dictate the wearable form that the device will become. Not only do the sensors matter but the materials used for the biosensors are also taken into consideration like biocompatible wearable sensors or even self-healing flexible wearable sensors. Currently, more biosensors are becoming miniaturized and wireless as technology is advancing. This will be discussed later in the report^25^.

## Different sensor designs

### Wearable sweat biosensor

Because skin covers so much of our body, skin-worn devices get a lot of attention in many types of wearable biosensors. Real-time analysis of biomarkers in biological fluids and real-time monitoring in several biomedical and fitness applications are possible using epidermal biosensors. Optical, electrochemical, and mechanical are common transduction modes used in skin-worn biosensors that bind to biocatalytic and ion recognition receptors^33–35^. Most of the reports in recent years have focused on electrochemical and colorimetric transduction methods that have led to significant advances in skin-wearable devices, allowing for easy sampling of epidermal bio-stream so that the wearer does not feel uncomfortable, such as the use of electronic skin or printed temporary tattoos. In addition, miniaturization of sensors into wristbands or embedding sensors into clothing ensures close contact with the skin while allowing the sensor to withstand the forces generated during body movement. Sweat glands are distributed throughout our bodies and are the most readily available biological fluid in chemical sensing applications. Sweat production can be achieved by exercise, or naturally in an environment with a relatively high body temperature, and then by pressure or electroosmotic stimulation^36^. In general, sweat contains various metabolites (such as lactate and urea) and minerals, and these biosensors perform a large amount of original humoral analysis for noninvasive monitoring of physiological health status and disease diagnosis management^37^. The epidermis is monitored noninvasively, which removes the issues associated with blood collection while still preserving the integrity of the protective stratum corneal skin. More research trials are needed, however, to show sweat's clinical utility as a diagnostic biofluid^36^. Because sweat analytes are mostly delivered into sweat via capillaries, establishing a clear and accurate link with blood concentrations is difficult. Here it is possible to monitor the rate change of sweat by simultaneously monitoring an analyte or skin impedance measurement of concentration distribution independent of sweat rate^35,38^. But there is also a problem here, the degree of dilution of the analyte during the excretion of sweat is affected by the rate of sweat and the rate of analyte distribution. Also, the epidermal biosensor has a target for measuring the concentration of analytes in the interstitial fluid (ISF). Within the active skin tissue of an organism, skin cells are surrounded by ISF, which provides nutrients, so there is a reliable correlation between blood and the concentration of ISF in many analytes. To evaluate ISF analytes in a non-invasive manner, these components must be extracted onto the surface of the skin, which can be done by reverse ion electroosmosis or ultrasonic electroosmosis. However, these methods are still flawed, and their accuracy will be affected by changes in extraction efficiency and skin surface contamination. Solving these problems requires advanced sampling methods and improvements to analyte monitoring methods^38^.

The epidermal wearable biosensor was first designed for detecting a single analyte for a variety of target analytes. This proof of concept is carried out using new compression-resistant materials and sensor structures to achieve a high degree of skin fit. This is critical for reliable and stable sweat sampling during exercise, such as electronic skin or temporary tattoos, in combination with screen-printed flexible electrodes, providing an appealing platform for skin-based biosensors^39,40^. As the skin has appealing electrochemical characteristics, it is ideal to design the electronic skin or temporary tattoos being in direct, continuous touch, and consistent flexibility with the base skin. Tattoo-based epidermal biosensors have now been shown to monitor critical sweat electrolytes and metabolites in real-time and non-invasively^41–43^. The first demonstrated continuous monitoring of lactic acid levels in sweat via an epidermal electrochemical biosensor, provides a dynamic real-time graph of an organism's lactate sweat during exercise^44,45^. Sweat lactic acid is a by-product of the metabolism of local sweat glands in the skin. When the organism undergoes vigorous exercise, it leads to a higher production rate of sweat lactate. Although lactic acid does not respond immediately to contemporary blood levels, it does indicate the amount of physical exertion consumed during long-term exercise and can be utilized as a metric of exercise efficiency without invasive blood sampling. The subject of study was requested to wear an electronically printed temporary tattoo biosensor in the study. Lactate oxidase is added to the sensor to measure the sweat lactate generated during exercise. The measurements confirmed that sweat lactate did indeed increase with the intensity of exercise^44^.

Great progress has been achieved in quantitatively analyzing sweat multiplexed sweat biosensor platforms based on a fully integrated chip wearable sensor array. Noninvasive multiplexed sensing is appealing, but accurate and dependable monitoring systems are required. By combining numerous sensing arrays, a team may simultaneously measure sweat metabolites, electrolytes, and skin temperature. This is a ground-breaking strategy that has aided the development of wearable sensors by bridging the gap between signal transduction, data processing, wireless transmission, and system integration, allowing for the processing and sharing of raw data. This advancement in the wearable sector is attributable to the use of flexible chip sensors and conformal circuit boards, which can be accurately evaluated using physiological state data signals processed during lengthy body movements. Multi-analyte sensing also detects human sweat and is used to calibrate the analyte signal of interest to increase physiologically relevant qualities. Because the sensor relies on the experimental embodiment of physical exertion to create perspiration, the actual effect of continuous monitoring will be influenced by restrictions, according to the systematic results of the experimental report^46^.

**Figure 3:**
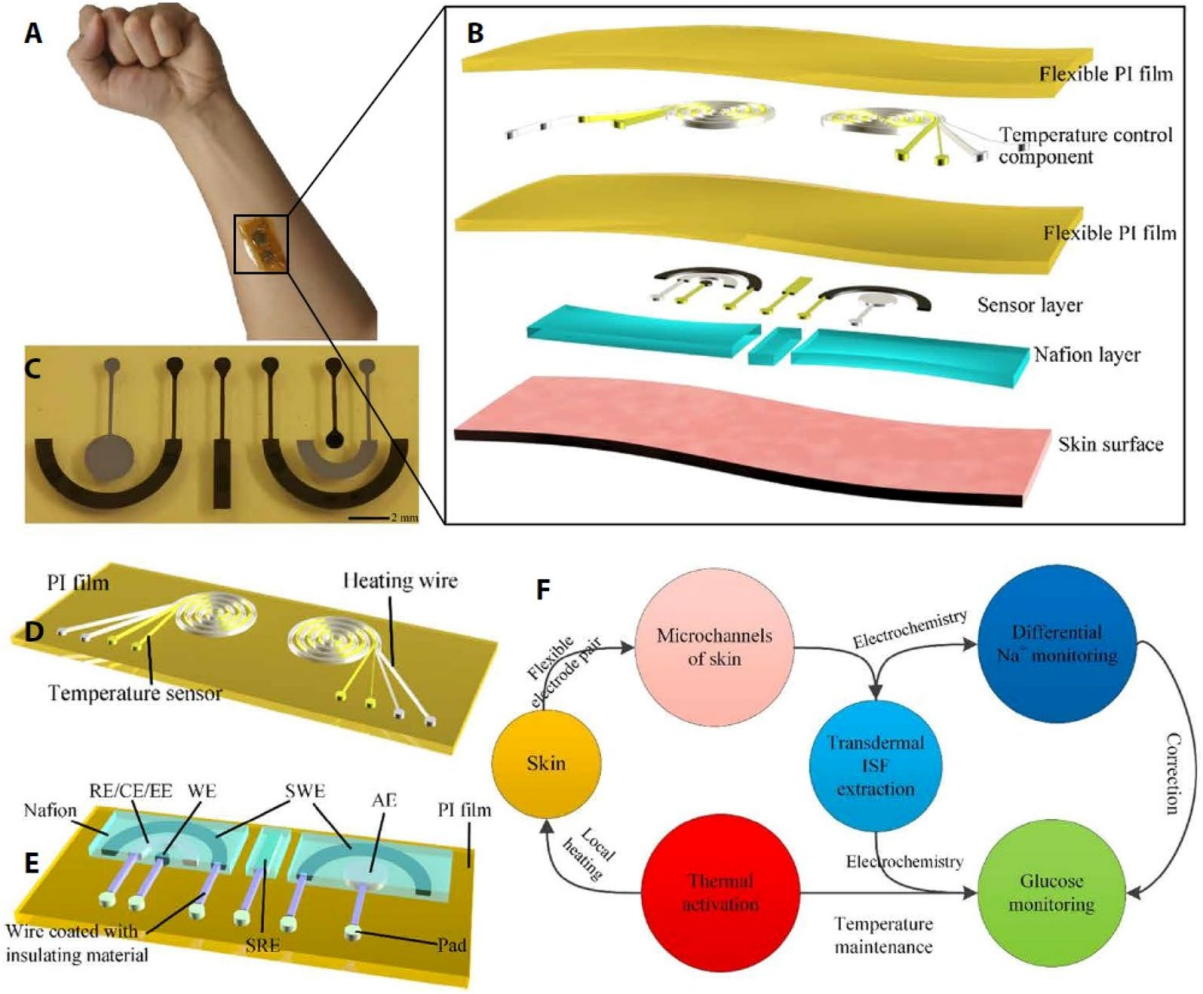
Flexible epidermal bio-microfluidic blood glucose continuous monitoring sensor^47^. (**A**) Proposed device. (**B**) The detailed structure of integrated epidermal biological microfluidic device. (**C**) Flexible glucose detection patch. (**D**) Temperature control component. (**E**) Glucose detection patch structure. (**F**) Working mechanism of integrated flexible epidermal biological microfluidic device.

Sweat glucose monitoring systems that combine pH, humidity, and temperature sensors have led to improvements in the therapeutic application of diabetes care. Currently, epidermal biosensors confront substantial obstacles in reliably measuring sweat glucose concentration, such as temperature variations and blood pH values affecting glucose concentration, as well as many sources of glucose contamination, irregular sampling rates, and low collection. Figure 3 shows a flexible epidermal sensor utilizing bio-microfluidics for blood glucose monitoring developed by Pu et al.^47^.

**Figure 4:**
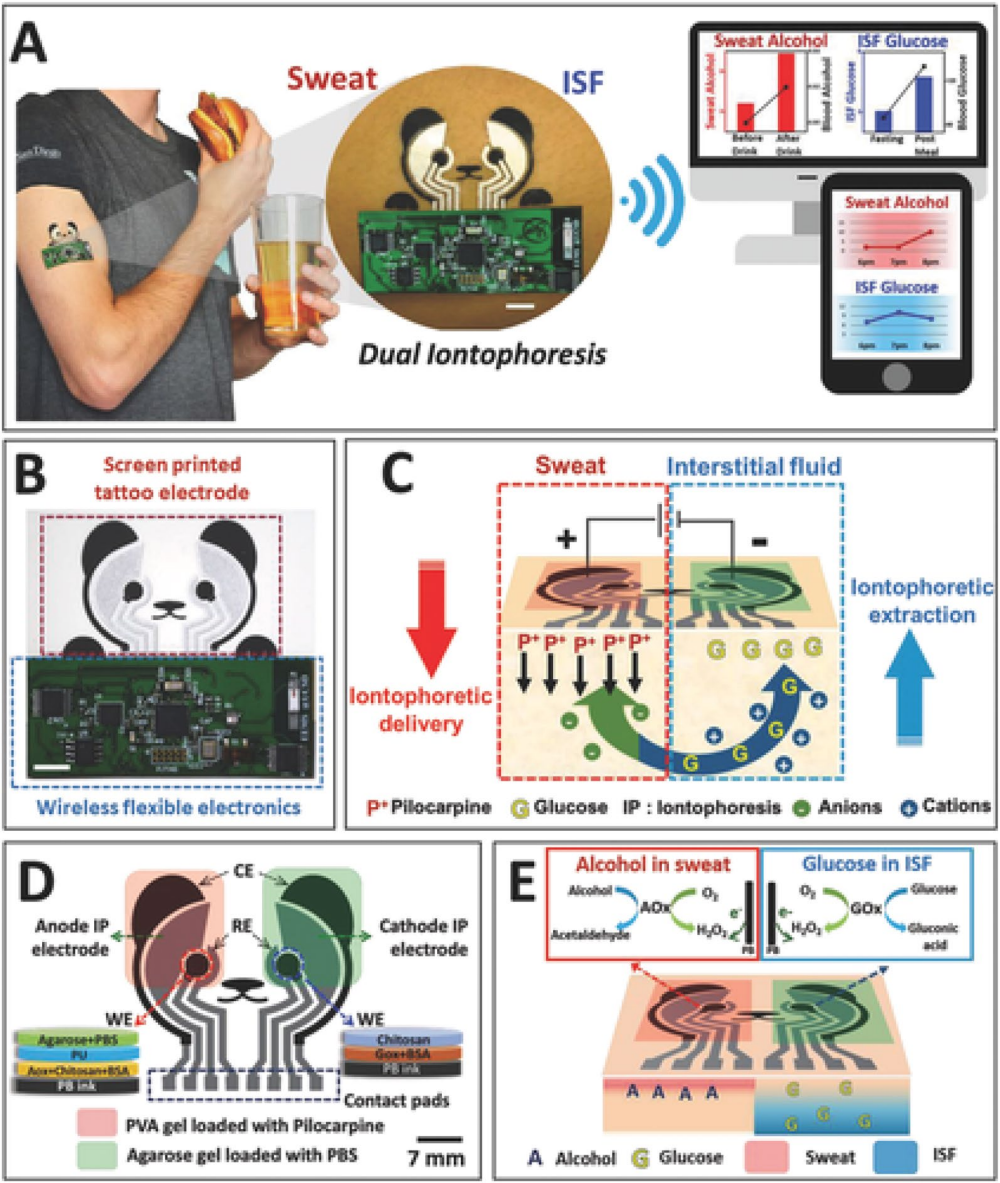
Epidermal iontophoretic biosensor^49^.

Much of this research has found a link between the concentration of glucose in sweat and the level of glucose in the blood, however, the accuracy of using sweat detection for threshold glucose level would need to be evaluated by further studies^48^. Functionalized graphene has been integrated onto flexible serpentine electrodes to improve the electrochemical bio-sensitivity of sweat glucose, according to research^41^. Although this operation can continually monitor and modify measurement results based on changes in immobilized enzyme activity to increase data accuracy, it can't overcome the problem of exogenous glucose sources interfering with the data. Figure 4 illustrates an iontophoretic biosensor that detects both sweat alcohol and glucose in the ISF developed by Kim et al.^49^. The electrode is a screen-printed tattoo fixed to flexible wireless electronics which utilizes iontophoretic extraction caused by the movement of cations from transdermal pilocarpine delivery. This development is able to sample two epidermal biofluids simultaneously using a cost-effective technique of screen printing and has shown promise in measuring sweat-alcohol and ISF-glucose levels.

Gao et al. demonstrates the efficacy of patch-based sweat biosensors in managing glucose levels therapeutically^46^. However, the report requires the wearer to exercise to monitor and evaluate blood glucose variations and effective and consistent real-time blood glucose monitoring. This is impossible when the wearer is not exercising. As a result, further research into this sweat monitoring gadget for diabetes management is required.

A new multiplexed wearable sensing approach that combines electrophysiological measures with biochemical marker data is currently being developed^46^. This technology uses a single sensor to monitor physiological chemistry and electrophysiology simultaneously, eliminating the requirement for separate physical and chemical sensors for detection. With the introduction of this novel wearable sensing approach, sensors can now take a significant stride forward in their multimodal development. Real-time optical monitoring of several markers and real-time sweat sampling with a microfluidic integrated colorimetric sensing device are examples of signal transduction techniques that can be used. The rapid collection of sweat, based on this technique, will not impair sweat collection owing to evaporation, and it will also not pollute the sweat collection target, overcoming the problem of traditional methods' inaccuracy and unreliability. Rogers Group has upgraded its epidermal pigment metric sweat-sensing microfluidics device to allow for multiple continuous sweat marker measurements^50^. A new skin-wearable flexible sweat sampling microfluidic flow.

system has also been developed by a research team^51^. This novel microfluidic sweat monitoring technique integrates fluorescent probes into skin surface systems to provide precise electrolyte measurements in real-time. This optical sensing fluid technology has a sensitivity that is comparable to that of established laboratory procedures^52^.

In addition, rather than just sensing perspiration or ISF, epidermal biosensors may also monitor and analyze the skin's surface. A bandage-style biosensor has recently been shown to detect tyrosinase on the skin's surface. The electrochemical equation of the benzoquinone product of the enzymatic reaction is used to determine the level of tyrosinase. This bandage-type biosensor can currently screen for melanoma swiftly, which bodes well for the healthcare monitoring sector^53^.

### Epidermal biosensor based on ion electrogenic therapy

In biological monitoring, ionic electrometry is used to get non-invasive epidermal biofluids^54^. An appropriate current is applied to the skin to guide ion migration induced by friction between the skin and the sensor. Cygnus, for example, has created a wearable wrist-worn system called the Glucowatch Biographer^55^. This system provided for noninvasive long-term blood glucose monitoring, but it was taken off the market in 2000. The reverse ion electroosmosis method has been observed to cause skin irritation, and its application necessitates a lengthy preparation period. Williams et al. provided a review on a new ionic electroosmosis platform that employs a flexible tattoo platform that conforms to the human body^56^. It considerably lowers the physical discomfort produced by reverse ion electrogenesis, and it is also easily imbedded in the skin's surface without making the wearer feel uncomfortable when exercising.

To capture more ISFs, a positive-charged hyaluronic acid is added into the wearable device, which dramatically improves glucose transport to the epidermis of the skin^57^. The glucose is extracted, processed with reverse ion electroosmosis, and then analyzed with a conformal glucose oxidase biosensor. This method greatly improves the rate of glucose extraction in the gap fluid and allows for more precise detection of data related to blood concentrations, implying that improved monitoring applications based on non-invasive reverse ion electroosmosis therapy may be able to overcome the limitations of previous reverse ion electroosmosis. However, the efficiency of glucose obtained by reverse ion electroosmosis is difficult to maintain, which may cause the volume of the sampling ISF to be inconsistent, impacting the change in glucose concentration.

A glucose monitoring patch based on graphene pixels with path selection has been developed in recent years to make the analyte collected by reverse ion electroosmosis more consistent^58^. Graphene offers excellent electrical and mechanical capabilities, as well as flexible lamination and ultra-thin transparency, and does not hurt human skin, making it a perfect material for this patch sensor. Graphene films were used in patches based on micropixel-based sensor arrays, and reverse ion electroosmosis was used to directly collect and measure glucose in the interstitial fluid of cells through hair follicles, allowing blood glucose levels to be monitored continuously and dynamically for a long time. The utilization of minuscule sensors in the array to monitor changes in blood glucose extraction inside and between skins through individual hair follicles is at the heart of the device, which also enhances measurement accuracy.

The development of epidermal wearable biosensors has allowed them to be used to detect a variety of medications, such as a sweat-based wearable sensor that uses pilocarpine's ionic electroosmosis or exercise to create sweat from which to detect caffeine. The sensing platform is based on current scanning for direct anode detection of caffeine. This sensing system has a lot of potential for monitoring drug-drug interactions in vivo, and it can be further developed and researched in the future^59^. Despite recent advancements, the current epidermal biosensor is still limited to the study of a single biological fluid.

### Tear-based wearable sensor

Tear fluid is another organism that may be used to assess physiological health, and biomarker molecules in tear fluid diffuse directly from the blood, indicating a positive correlation with blood pressure marker concentrations. As a result of the analysis of tear fluid, it allows for the identification of eye problems. Tear fluid is a component of the eye's anti-fouling mechanism, and its composition is significantly less complex than blood's, making it a great candidate for non-invasive monitoring and diagnosis^60–64^. Tear fluid is primarily produced by lacrimal glands and conjunctival cup cells with the main component of tear fluid being protein, along with ions, glucose, and urea. It's worth noting that the glucose concentration in the tear fluid released by the tear glands is significantly associated with the blood glucose level. However, if the eye is subjected to external stimuli, and tear fluid is released in an aberrant manner, this link can be broken.

Because the sample size of the diagnostic tear sample is small, and it will be accompanied by evaporation during the collection process. The generation of individual tear fluid changes throughout the day as well as the difficulty of the collection method; it is very easy to affect the concentration of markers in the sampled tear fluid. As a result, the technique of collection, which is most typically a glass capillary or a Schirmer’s strip, has a significant impact on the accuracy of this in vitro tear diagnostic assay^65^. Reflex tears are tears produced in response to emotional or mechanical stimuli, and they differ from tears produced by natural secretions in terms of composition. The relevance of developing wearable tear sensing platforms that do not require eye stimulation is highlighted by these developments and obstacles.

Because contact lenses can be worn without causing discomfort to the eyes and can always keep in direct touch with the tears shed by the eyes, contact lens-based devices are appealing for resolving tear collecting^66–69^. Incorporating all the requisite biosensing, data processing, and power supply into contact lenses, on the other hand, is a big difficulty in contact lens design. Due to the rapid development of various materials for contact lens manufacturing, such as soft materials, a high degree of flexibility can be provided to minimize wearing discomfort caused by eye irritation while also providing the required oxygen permeability, improving the accuracy of continuous monitoring of tear glucose or metabolites. Simultaneously, another new method is proposed: using holographic contact lenses to quantify the potential of glucose in human tears in the physiological environment. This method has the advantage of not requiring power and the data is easy to read, allowing for continuous signal monitoring^70^.

**Figure 5:**
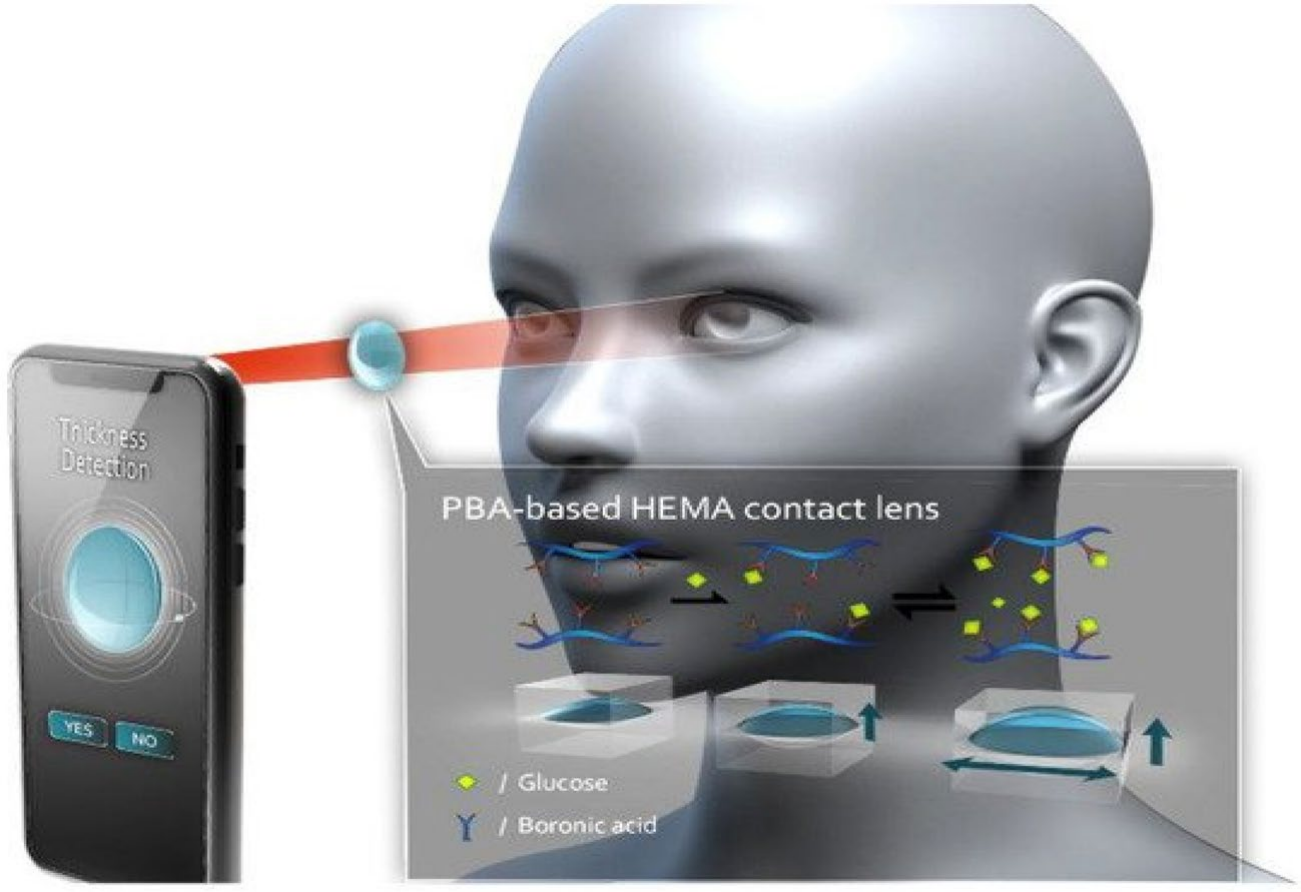
Tear-based wearable biosensors^72^.

The University of Washington's Department of Electrical Engineering's research team has made more progress in this area. The researchers investigated various biosensing methodologies to obtain high sensitivity and data accuracy, which allowed them to tackle the interference problem by using a dual sensor configuration. The device is further enhanced by the inclusion of a 2.4 GHz-based wireless read chip that employs far-field electromagnetic radiation^71^. Figure 5 illustrates a contact lens that monitors glucose levels developed by Lin et al.^72^. This development works by a reversible covalent interaction which alters the thickness of the contact. This thickness is then read by the camera of a smartphone and presents a noninvasive measurement for glucose levels utilizing glucose sensing via chemical interactions.

Over years of hard effort, the Google research team has made great progress in their separate areas of electronic downsizing and applied medical technology, establishing a contact lens platform for tear glucose monitoring in conjunction with Novartis^73^. A wireless control chip, a microelectrochemical converter, an antenna, and an embedded hydrogel skeleton for non-invasive glucose measurement of surrounding tears are all included in this soft contact lens platform idea product. However, given the delays in clinical trials and subsequent commercial releases of this product, developing a high-performance technology platform based on contact lenses poses considerable obstacles.

By merging glucose contact lens sensors and intraocular pressure contact lens sensors using wireless technology^74,75^, a research team from the National University of Science and Technology of Ulsan in South Korea has developed smart contact lenses for wireless eye diagnosis. Despite the device's ability to multiplex sensing, the simultaneous operation of the two sensors has yet to be confirmed. Interference and biocompatibility between the two sensors will be thoroughly examined in future human trial investigations. Later, in stealth biosensor research, the researchers used integrated wireless display and wireless power transmission circuitry to enable the real-time display of the in vivo glucose response in rabbit tears. This cutting-edge technology is made of transparent and soft materials that ensure the wearer's comfort without obstructing eyesight. It requires no additional power supply because it is a wireless power module. However, further research is needed to show the feasibility of sensing performance in the human body and its capacity to track glucose fluctuations throughout the day.

A photon microstructure sensor was put on commercially available contact lenses by a research team at the University of Birmingham in the United Kingdom^76^. The smartphone records the varying reflected powers, which correlate to changes in tear glucose. This technology has been proven to respond to glucose quickly and accurately. This capacity makes it perfect for replacing electrochemically based contact lens biosensors, overcoming power supply and data transmission problems in tiny devices. A small electrochemical sensor that resembles a spring is worth mentioning in addition to the contact lens platform. Multiple spiral electrodes are coated with a protective polysaccharide-based hydrogel substance in this NovioSense-designed sensor. A continuous conduit of tear fluid is provided by inserting the device under the conjunctival vault of the eyeball. It does not cause discomfort to the wearer because it is positioned at the bottom of the eye, beneath the eyelids, and wireless data transmission can be utilized to continually detect tear glucose. It was found in a clinical trial that there was a robust link between tear glucose and blood glucose levels^77^.

### Saliva-based wearable biosensors

Saliva has been more popular as a diagnostic fluid in recent years as the number of oral disorders has increased. Many biomarkers in saliva enter saliva by direct cell-to-cell transfer in the human body, allowing saliva to represent the physiological condition of the human body and serving as an ideal noninvasive sampling method to replace blood analysis. Saliva is easy to collect and has a high protein concentration, making it ideal for biomarkers that will be used to monitor disease and stress in biomedical and health settings^78–82^.

Saliva is made up of three pairs of big salivary glands and a slew of small buccal glands scattered around the oral mucosa's surface. Saliva is a colorless, clear, viscous liquid with a mildly alkaline pH that is primarily made up of water with a little quantity of inorganic and organic materials. There are many kinds of inorganic compounds, and the main components are potassium ions, sodium ions, chloride ions, and bicarbonate ions, with potassium ions and bicarbonate ions having the highest concentrations. Although some of these saliva biomarkers can provide useful diagnostic information for clinical trials, there is currently limited study on dental wearable biosensors due to the large protein population in saliva and the risk of biocontamination from low quantities of biomarkers^83–87^. Despite the difficulties, oral biosensing platforms may gather dynamic chemical information from saliva in a non-invasive manner, which is an appealing method.

It is believed that the first wearable oral sensors were exhibited in the 1960s. It works with a topical denture platform to track chewing, plaque pH, and fluoride levels. However, it necessitates the sensor replacement of numerous teeth, and the internal sensor may cause intraoral leaking and is not widely used. The Princeton University study team improved on this concept by printing graphene nanosensors on water-soluble silk threads and transferring them directly to tooth enamel to enable passive and wireless monitoring of microorganisms, advancing oral biosensing technology research^88^. This idea product is intended to monitor bacteria on the teeth remotely, but it can also be used to monitor other saliva biomarkers^82,88–92^.

In vitro investigations have revealed a strong correlation between blood and salivary metabolite levels, paving the way for the creation of current oral saliva metabolite sensors, notably the link with wearing dental goggles^93^. By incorporating screen-printed enzyme electrodes into the device, a research team from the University of California, San Diego's Department of Nanoengineering produced an electrochemical biosensor of saliva metabolites (mainly lactic acid) in the form of a tooth guard^82^. Salivary lactate is closely related to blood lactate and can be used to determine physiological response and performance. Using lactase oxidase, the gadget can detect salivary lactate selectively. Electro-polymerization of o-phenylenediamine protects sample targets from contamination in undiluted saliva, allowing for continuous and noninvasive monitoring of the organism's health.

Another team also developed a uric acid biosensor in the form of a tooth guard that measures uric acid levels in saliva, allowing for noninvasive indirect monitoring of blood uric acid levels^89^. Uric acid in the blood is a biomarker for several conditions, including hyperuricemia, gout, and renal syndrome. This platform has demonstrated sensitivity, specificity, stability, and speed of reaction. We may now acquire dynamic chemical data on oral saliva indicators because of this. While these dental biosensing devices are appropriate for fitness or diagnostic applications, more standalone platforms are needed to broaden the variety of applications, such as continuous glucose monitoring in daily life.

**Figure 6:**
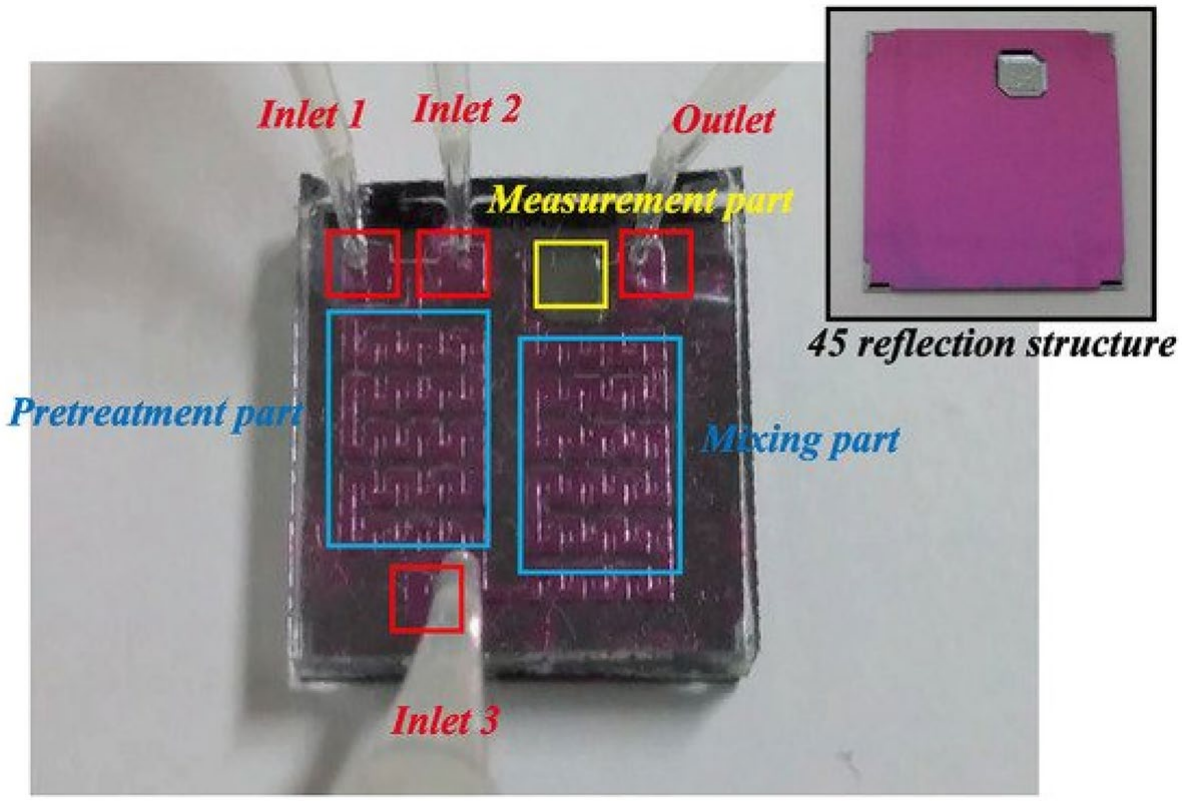
Saliva-based LOC biosensor^96^.

A tiny and removable "cavity sensor" has been developed by multinational research teams from Japan and the United Kingdom^94^. The sensor surface is constructed of a GOx modified polyethylene glycol polymer with an integrated wireless transceiver installed on a bespoke monolithic tooth guard that fits the contour of the wearer's teeth for sensing salivary glucose. The strong relationship between blood glucose and salivary glucose makes glucose sampling a relatively convenient and accessible option. Before micro-wearable platforms may be considered for screening or monitoring diabetes via salivary glucose, more large samples of human research are required. Kacheon University in South Korea recently showed a dental platform-based wearable sensor^95^. Introducing biocompatible materials and RF sensors into the teeth to assess alcohol level, salt, sugar, pH, and temperature in saliva allows for wireless food monitoring while feeding. To achieve accuracy, the approach also necessitates a thorough examination of biomarker directional selectivity. Figure 6 illustrates a lab on a chip (LOC) based optical sensor that measures glucose in saliva. Although this fabricated model requires multiple components; it shows the feasibility of extracting a glucose measurement from saliva. This model also uses a photodiode and a light-emitting diode to obtain a light absorbance value which relates to glucose concentration^96^.

A coarse cellulose membrane is produced on the glucose sensor as an inhibitory interference membrane in the sensor project research, allowing glucose to be measured in saliva^90^. This eliminates the requirement for any preparation of human saliva, allowing for accurate measurement of glucose in saliva in the human body. Another oral monitoring gadget uses ultra-thin, retractable electrical devices and small sensors to enable remote wireless telemetry sodium intake^97^. Human experiments have shown that real-time salt consumption monitoring is possible, which is exactly what is required to treat hypertension. Naturally, the device's toxicity is now only assessed in the absence of a chemical sensor layer. A thorough study of the recognition layer's biocompatibility is required for practical oral applications. Overall, major studies of oral sensing platforms are still needed to ensure their safety and reliability in real-world applications. Equipment safety, as well as minimizing surface contamination produced by other saliva components and food crumbs, requires specific attention.

## Machine learning applications in wearable devices

Health monitors worn by consumers could save lives in the future. But how do you tell the difference between a crisis and a normal state in an emergency? Because the sensor has the potential for false positives, if there are too many people wearing it, nearby hospitals may frequently receive false positive information, wasting valuable medical resources. Rejab's paper from 2014^98^ mentioned an approach that can guarantee a high degree of sensitivity while lowering the risk of false positives. Because the sensor can be continuously monitored and the sample data is sufficient, effective weights can be trained to evaluate the patient's condition, reducing the possibility of false positives, he stated that it is necessary to generate a similar general model based on the patient's underlying disease, and then train the sensor data through support vector machines, such as LASVM, ISVM, and K-prototypes clustering. It is possible that machine learning can not only filter out false signals, but also create artificial neural networks to train the data based on various types of sensor data to determine the health state of users.

Machine learning is crucial in the data processing and sensor modelling processes. Stetter's study focuses on evaluating external knee flexion and adduction moments in a variety of sports using machine learning and wearable sensors^99^. This approach, which is based on joint torque modelling and the position of wearable sensors, can successfully assist knee osteoarthritis (KOA) patients in evaluating their own exercise intensity^100^. Stetter's study included 13 volunteers who had two inertial sensing measurement units (IMUs) attached on their right thigh and calf while doing six different motor activities. The two sensing units can gather two sets of data at the same time: the external knee flexion moment (KFM) and the external knee adduction moment (KAM) (KAM).

Stetter creates a collection of physical models for artificial neural network (ANN) training based on IMU inputs, whole-body kinematics, and ground reaction forces. The findings of the trials show that using only two IMUs can provide a helpful biofeedback system for KOA patients to a limited extent, and that the IMU signal was low-pass filtered by Stetter (zero-phase Butterworth fourth-order filter). Then, the signal is input into the ANN model for training. After 1000 iterations of ANN training, the different weights of the five action tasks are obtained according to the maximum correlation between KFM and KAM. This allows the patient to get more accurate feedback after the relevant data is passed through the model again^99^.

However, Stetter's research did not specify the artificial neural network's training approach but based on the maximum correlation of the final training result, it may be assumed that the clustering algorithm and unsupervised learning were utilized. Stetter's training method has a potential flaw; unsupervised learning can be employed if there are too many neurons, but with only five action tasks, two IMU signals, and thirteen persons, this does not imply a massive neural network. Meta, on the other hand, does not require unsupervised learning. The data of thirteen people can be taught in batches, the neuron input layer is made up of the two IMU signals from one person's five action tasks, the hidden layer is a multi-vector machine (MLP), and the training method is supervised learning or reinforcement learning. You can receive more useful data with weights, not simply the most relevant data from the clustering method.

## Discussion

### Major challenges for designing sensors

Wearable sensors are currently hampered by several technical issues^79,92,101^. Human–computer interaction, intelligent sensing, and flexible electronics are all inextricably linked to the advancement and spread of sensing technology as the core technology of wearable devices. The size, quality, power consumption, dependability, and stability of sensors are critical for the user experience, wearability, and power consumption of wearable devices, among other things. Power supply, communication security, and privacy are also major concerns.

It's vital for wearable sensors to be accurate and trustworthy for them to be accepted in the market. The surface contamination effect, which is the key factor impacting the sensor's ongoing operation, has a significant impact on the accuracy of wearable biosensors. Robust anti-pollution surface protection, as well as dynamic calibration techniques including multimode, multi-marker sensing, and drift correction, are required to assure long-term reliability on the body.

Concerns like hardware, power supply, and communication challenges are crucial to the practical deployment of these sensing devices, and they are not restricted to wearable biosensors. Hardware components must be tightly linked with biosensor platforms and customized to meet the specific needs of the application. Because of their versatility and cost-effectiveness, printed wireless circuit boards with full-featured microcontrollers are commonly employed in wireless platforms. This printed circuit board can be connected to the battery in a variety of ways.

Another important criterion for wearables is minimal power consumption during continuous monitoring to offer the wearer or other end users with valuable and timely chemical information. When high sampling frequencies are necessary, this may necessitate a trade-off between energy usage and data rate. The importance of efficient data processing and effective and secure data exchange cannot be overstated. A lithium-ion or alkaline battery is the most popular means to power a wearable biosensing platform. They are, however, bulky and can cause toxicity issues, particularly in lithium-ion systems. Batteries are currently being made using flexible materials to improve wearability; however, it has yet to be demonstrated that the energy density is sufficient for long-term use.

### Major applications and applicational limitations

Oral biosensors that are based on saliva are likely to be heavily contaminated. Saliva includes far more proteins than noninvasive bodily fluids like perspiration or tears because it contains complex components. As a result, surface protective coatings for oral biosensors must be prioritized. To decrease the impacts of biological contamination and to eliminate simultaneous electrically active interference, sensor coating materials should be carefully chosen. At the same time, enzymes are applied to the sensor's surface to prevent the release of potentially harmful components.

Wearable biosensors, unlike traditional lab-based biosensors, can compromise the stability of fragile biosensors when exposed to long durations of outdoor activities in an uncontrolled environment. Multiplexed sensing technology, which includes biosensors and physical sensors, allows for active temperature, pH, and humidity calibration. In addition to potential contamination from the surrounding environment, mixing with stale bodily fluids, and continuous signal drift involving the calibration of the associated sensors, accurate measurements worn on the body necessitate careful attention to potential contamination from the surrounding environment, mixing with stale bodily fluids, and continuous signal drift involving the calibration of the associated sensors. Using proper microfluidic sampling methods and improving surface coating techniques can help address some of these issues.

Traditional micro supercapacitors have a "sandwich" stacked structure that makes them unsuitable for application in wearable devices, particularly flexible wearables, due to issues such low flexibility, lengthy ion diffusion distances, and complicated integration. Cheng et al. developed a solution to use a novel equipment architecture and integration technique to overcome the problem of micro supercapacitor stretching and bending in a snake-shaped island bridge configuration, resulting in a new micro supercapacitor array. The conductivity and amount of charged ions absorbed are improved by using a 3D laser to produce graphene foam^102^.

Smart watches, fitness wristbands, sleep trackers, and other smart health monitoring devices that are popular today are not safe, accurate, or intelligent, and do not provide a high degree of service to users. There are numerous security, reliability, and other challenges with these IoT portable sensors. We know from the usefulness that the altimeter, ambient light sensor, accelerometer, optical heart rate monitor, and other sensors currently advertised for smart bracelets that can be used for sleep monitoring are altimeter, ambient light sensor, accelerometer, optical heart rate monitor, and so on. The user's physical condition is assessed and based on the dark environment and low-amplitude motion, the user's time in bed and wake-up time are calculated. Tabia et al. conducted a study using data from the Fitbit blaze fitness tracker. They discovered that the daily data of the smart bracelet is independent of the data of the preceding and subsequent days after 8 months of data analysis^40^.

Inconsistent sleep quality measurements occur regularly and there is no way to create a learning model in this situation. This is because the sensor data is insufficient, and there is no way to acquire reliable sleep data using EEG. As a result, relying alone on the smart wristband to monitor sleep is incorrect, and relying solely on the smart bracelet will result in the loss of some data, i.e., the generation of incorrect data and the loss of right data.

A long-term dark environment and a stable body will cause the bracelet to believe that the user is sleeping at this time, but this is not always the case, such as in a movie theatre, where a long-term dark environment and a stable body will cause the bracelet to believe that the user is sleeping at this time. In current studies, each day's sleep is strongly related to the next few days, allowing users to predict if today's sleep has improved based on the prior day's sleep using machine learning models^103^.

Smart bracelets, on the other hand, have such a high level of uncertainty that their learning model is unreliable and untrustworthy, and some smart bracelets do not even use artificial intelligence. This distinguishes the sleep data obtained by the user from the sleep data tracked by PSG, as described by Kwon^104^. As a result, the user can only get a rough idea of how much sleep he or she gets, but no proper health advice. As a result, the smart bracelet cannot function as a true wearable health sensor.

Regarding power supply, wearable supercapacitors provide quick charging and discharging characteristics, as well as a low weight and energy density capacity. Some wearable power supply will collect energy to charge during the wearer's exercise, depending on the charging type of the wearable platform. Solar energy, the movement of devices based on piezoelectric or electrostatic principles, heat generation of thermoelectric materials, or the chemical composition of sampled biological fluids to power wearable biological fuel cells are all possibilities for wearable batteries. Wearable biofuel cells have promise as a source of electricity for non-invasive wearable platforms. It can be utilized as a self-powered biosensor and can harvest energy from the same biological fluids, but its stability is uncertain at this time. Wearable power supply advancements are critical, especially as the demand for power from multiplexed sensing platforms grows. It can be compensated for via adaptive algorithms that reduce energy demand, in addition to the development of powered and more energy-efficient equipment.

### Future directions for sensors

Researchers are currently attempting to build more advanced sensors to make wearable gadgets with more humanized functions in response to the power supply problem noted above^105–108^. A self-powered stretchy health monitor constructed of graphene material is one of the wearable sensors developed by researchers at Pennsylvania State University in the United States. Although self-charging power supply units for stretchable energy harvesters already exist, Cheng et al. claims that they are costly to make, cumbersome to transport, and have "poor and unsteady output power"^102^. Currently, stretchable conductors, semiconductor materials, and devices, as well as self-healing and biocompatible materials, are widely used in electronic skin physical sensing platforms, and much-related research is focused on the fusion of nanomaterials and MEMS processes. Carbon nanotubes, graphene, and other nanomaterials are examples.

The island bridge design created by Cheng's team employed non-layered ultra-thin zinc phosphide nanosheets and 3D laser-induced graphene foam, which not only allowed for efficient charge and discharge, but also improved the tensile properties of the micro supercapacitor array^102^. Cheng's team's development of the self-powered wearable relies heavily on the micro supercapacitor array. Because there is no chemical reaction between energy storage and release, supercapacitors with more than 100,000 charge and discharge durations are widely anticipated in the field of self-power, and many applications aspire to use supercapacitors to replace ordinary lithium batteries.

Another challenge is that most wearable biosensors currently only assess a small number of biomarkers. In the future, the industry should work to develop novel biosensor formats and improve non-invasive biosomal fluid sampling to monitor a larger range of biomarkers. Understanding the composition of each organism's body fluids, as well as their relationship to blood chemistry and specific medical illnesses, is crucial to gaining general clinical adoption of wearable technology in healthcare. A major indicator of its detection is the real-time correlation of marker levels in noninvasive humoral sampling with concurrent marker concentrations in the blood. In the actual world, rigorous and repeatable interpretation of biosensor results is also a goal, especially in applications that may require a clinical or operational reaction.

To identify novel biomarkers in the future, systematic and in-depth examination of the composition of each organism's body fluids will need to be performed, which has never been done previously in the context of wearable sensor research. Noninvasive testing can be expanded beyond the detection of a small number of metabolites and electrolytes, for example, by employing noninvasive immunoassays to assess a variety of protein disease indicators, hormones, and stress markers. Similarly, in addition to existing fluid types, potential from novel fluid types should be explored (urine, mucus, and semen). Other fields of biomedicine, such as the clinical development of new experimental medicines guided by biomarkers, will benefit from this real-time study of a wider spectrum of biomarkers.

To detect very low concentration biomarkers, wearable immune sensors require complex microfluidic devices with several stages and extended reaction times. It streamlines label-less detection techniques and has a lot of potential in healthcare, fitness, and a wide range of biocontainment applications. While most wearable devices focus on a single parameter, efforts should be made to monitor a wide range of biomarkers simultaneously and noninvasively. This more complete analysis not only allows for a more thorough examination of physiological states, but it also allows for dynamic calibration and correction of responses for more precise monitoring. Biosensors with multiple sensing methods for the same analyte can also be more reliable.

In the future, smartphone identification of mentally healthy patients will become more comprehensive, mainly thanks to reinforcement learning and self-supervised learning. For patients, a learning model will be continuously upgraded, generating personalized data based on the labels provided by the patient, which will give accurate answers through the doctor's recommendations.

The pacemaker through the radio frequency signal can effectively notify the clinic to give first aid to the guests, and through the machine learning algorithm, the misjudgment of the pacemaker can be reduced. In the future, according to the current technology, the pacemaker can work together with multiple sensors. Atrial fibrillation can be identified not only by the pacemaker, but also by the heart rate sensor and galvanic skin sensor of the smart bracelet. The inference is that when an artificial neural network is modeled for this, the other sensor can provide an emergency signal to the pacemaker when one of the sensors does not work. This can more safely ensure the health of the user.

## Conclusion

Wearables have been found to decrease sedentary behavior and increase overall health in users. Many companies in industrialized nations are already using wearable sensors to motivate patients to participate in rehabilitation programs and daily activities. Obesity and diabetes are two chronic diseases that can be prevented by living a healthy lifestyle. Furthermore, using wearable sensors to monitor patients can assist individuals and medical professionals in making shared decisions regarding future treatment plans. The artificial neural network will be trained and learned from the patient's bodily data, and the proper information will be given to medical professionals, which will be a key tool for encouraging patient adherence to physician prescriptions.

Patients can undertake self-data monitoring and potentially ameliorate symptoms of chronic diseases using wearable sensors for health, which can help them enhance their self-control. Patients, for example, can collect their own motion data on their cellphones using sensors such as light sensors, gyroscopes, and accelerometers, and then use software apps to diagnose and counsel themselves on mental illness. The sleep situation may be obtained using the smart bracelet and patch sensor based on the EEG, accelerometer, heart rate sensor, and light sensor, and then the sleep situation of today can be compared to the sleep situation of yesterday, and the patient's health state can be compared.

Wearable gadgets with real-time positioning, voice aid, and emergency assistance for Alzheimer's patients can considerably boost their independence, allowing medical staff to operate in multiple lines and increase efficiency. Patients with Alzheimer's disease can also free up more space with real-time positioning and voice help, as well as have emergency rescue functions without risking their lives. Simultaneously, there are numerous chronic disease signs that are difficult to identify in everyday life. Patients with mental illness and heart problems, for example, can receive input via the smart bracelet's heart rate sensor. In the future, there will be further development. Healthy living is an important development direction for wearable sensors.

People will trust smart gadgets with sensors if data transmission and privacy are ensured. Sensors will enable basic diagnosis and illness intervention for people in the future, making medical resources accessible to all. When consumers receive data from wearable sensors for health and believe that the feedback is of reference value, the patient's undiagnosed conditions can be targeted with appropriate interventions.

Wearable devices, because of the extensive application of materials, have profited greatly and developed swiftly because of the development of many technologies, but there are still certain obstacles to be overcome, and there is a lot of potential for progress. First and foremost, there is an issue with sweat detection equipment because it cannot be reused, and by increasing the outflow system, the equipment may be recycled. Second, while most wearable devices can detect continuously for minutes to hours, they cannot meet long-term wear requirements, so a superior combination of supercapacitors and other capacitive devices can be used to build a smaller and more durable power source. Wearable gadgets also have a lot of components, which might impair comfort while wearing them, and biocompatibility is another factor to consider. Wearable devices can increase their intelligence and lower their size to make them more pleasant to wear while integrating the natural biocompatibility and biodegradability of biomaterials to solve their safety difficulties, thanks to the development of wireless transmission and novel materials. Finally, while wearable device detection is currently limited to physiological health detection, as materials science, nanotechnology, communication technology, and biotechnology advance, it will become increasingly important to synthesize sensing elements with novel properties to broaden the detection range of wearable devices.

In conclusion, while wearable devices still have some issues, with the continued development of synthetic materials and detecting methods, as well as the progress of detection platforms and transmission technologies, wearable devices will have a brighter future.

## Data Availability

All data generated or analyzed during this study are included in this published article.
